# Supplementary material for: Influence of Perceived Maternal Self-Efficacy on Exclusive Breastfeeding Initiation and Consolidation: A Systematic Review
Source: Healthcare (Basel). 2024 Nov 24;12(23):2347. doi: 10.3390/healthcare12232347 (PMC11641614; doi:10.3390/healthcare12232347)
Supplement: Supplementary file 1 [file healthcare-12-02347-s001.zip › Supplementary Table S2.pdf]

Supplementary Table S2. Evaluation of the quality of each study (Argumedo et al., 2017). [28]

**Research question:** *Is the study based on a clearly defined research question?*	Yes	No	Partially	No information
**Method:** *Did the study method minimize bias?*	Yes	No	Partially	No information
**Results:** Are the results correctly synthesized and described?	Yes	No	Partially	No information
**Conclusions:** *Are the conclusions justified?*	Yes	No	Partially	No information
**Conflict of interest:** *Is the existence or absence of conflict of interest well described?*	Yes	No	Partially	No information
**External validity:** Are the results of the study generalizable to the population and context of interest?	Yes	No	Partially	No information

	**Method YES**	**Method PARTIAL**	**Method NO**
**Majority of other criteria YES**	High Quality	Medium Quality	Low Quality
**Majority of other criteria PARTIALLY**	Medium Quality	Medium Quality	Low Quality
**Majority of other criteria NO**	Low Quality	Low Quality	Low Quality
