# Supplementary material for: Influence of Perceived Maternal Self-Efficacy on Exclusive Breastfeeding Initiation and Consolidation: A Systematic Review
Source: Healthcare (Basel). 2024 Nov 24;12(23):2347. doi: 10.3390/healthcare12232347 (PMC11641614; doi:10.3390/healthcare12232347)
Supplement: Supplementary file 1 [file healthcare-12-02347-s001.zip › Supplementary Table S3.pdf]

Supplementary Table S3. Quality assessment corresponding to each article.

**Author, (year)**	**Research question**	**Method**	**Resul ts**	**Conclusions**	**Conflict of interesest**	**External validity**	**Quality**
Noel-Weiss,J. et al., (2006) [29]	Yes	Yes	Yes	No	No	No	Medium
Awano, M & Shimada,K. (2010) [30]	Yes	Partially	Yes	Yes	Yes	No	Medium
McQueen, K.A. et al., (2011) [31]	Yes	Yes	Yes	Yes	Yes	Yes	High
Ansari,S. et al., (2014) [32]	Yes	Partially	Yes	Yes	Yes	Partially	Medium
Glassman, M.E. et al., (2014) [33]	Yes	Partially	Yes	Yes	Yes	No	Medium
Otsuka, K. et al., (2014) [34]	Yes	Yes	Yes	Yes	No	Partially	Low

Wu, D.S. et al., (2014) [35]	Yes	No	Yes	Yes	Yes	No	Low
Henshaw, E.J. et al., (2015) [36]	Yes	Yes	Yes	Yes	Yes	Partially	High
Chan,M.Y. et al., (2016) [26]	Yes	Partially	Yes	Yes	Yes	Partially	Medium
Ip, W.Y. et al., (2016) [38]	Yes	Yes	Yes	Yes	Yes	Partially	High
Araban,M. et al., (2018) [39]	Yes	Yes	Yes	Yes	No	Partially	High
Shariat,M. et al., (2018) [40]	Yes	Yes	Yes	Yes	Yes	Partially	High
De Roza, J.G. et al., (2019) [41]	Yes	Yes	Yes	Yes	Yes	Partially	High
Tseng, J.F. et al., (2020) [42]	Yes	Yes	Yes	Yes	Yes	No	High
Vakilian, K. et al., (2020) [43]	Yes	Yes	Yes	Yes	Yes	Partially	High

Wu,S.F.V. et al., (2021) [23]	Yes	Yes	Yes	Yes	Yes	Partially	High
Wong, M.S. & Chien,W.T. (2023) [45]	Yes	Yes	Yes	Yes	Yes	Partially	High
Yesil, Y. et al., (2023) [46]	Yes	Yes	Yes	Yes	Yes	Partially	High

Note: Source: Prepared by the authors. From: Lopez de Argumedo, M.; Reviriego, E.; Gutierrez, A. Updating of the Shared Work System for Systematic Reviews of the Evidence and Critical Appraisal (FLC 3.0 Platform). Ministry of Health, Social Services and Equality. Technology Assessment Service of the Basque Country. 2017. Available online: https://www.ser.es/wp-content/uploads/2018/04/Informe-OSTEBA-FLC-3.0.pdf
